# TRIM3 inhibits P53 signaling in breast cancer cells

**DOI:** 10.1186/s12935-020-01630-z

**Published:** 2020-11-23

**Authors:** Xinxing Wang, Yujie Zhang, Xinhong Pei, Guangcheng Guo, Bingjian Xue, Xin Duan, Dongwei Dou

**Affiliations:** Department of Breast Surgery, The First Affiliated Hospital of Zhengzhou University, Henan Province 450052, Zhengzhou, People’s Republic of China

**Keywords:** TRIM3, P53, Breast cancer, Ubiquitin

## Abstract

**Background:**

Beast cancer is the most common women cancer worldwide, while two third of them are ER alpha positive breast cancer. Among the ER alpha positive breast cancer, about 80% are P53 wild type, indicating the potential tumor suppression role in ER alpha positive breast cancer. Since P53 is an important safeguard to inhibit cell malignant transformation, reactivating P53 signaling could a plausible approach to treat breast cancer.

**Methods:**

TRIM3 protein levels were measured by western blot, while the P53 classical target genes were measured by real-time PCR. WST1 assay were used to measure cell proliferation, while cleaved caspase-3 was used to detect cell apoptosis. Protein stability and ubiquitin assay were used to detect the P53 protein ubiquitin and stability. The immuno-precipitation assays were used to detect the protein interactions. Immuno-staining was used to detect the protein localization of P53 and TRIM3, while the ubiquitin-based immuno-precipitation assays were used to detect the specific ubiquitination manner of P53.

**Results:**

In our study, we identified TRIM3 as an endogenous inhibitor for P53 signaling. TRIM3 depletion inhibited breast cancer cell proliferation and promoted apoptosis. In addition, TRIM3 depletion increased P53 protein level in breast cancer cell. Further investigation showed that TRIM3 could associate with P53 and promote P53 K48-linked ubiquitination and degradation.

**Conclusion:**

Our study identified a novel post-translational modification mechanism between TRIM3 and P53. TRIM3 depletion or blockage could be a promising strategy to rescue P53 signaling and inhibit breast cancer progression.

## Highlights

1. TRIM3 facilitates breast cancer cell growth and anti-apotosis.
2. TRIM3 inhibits P53 protein and its signaling activity.
3. TRIM3 interacts with P53 and promotes P53 K48-linked ubiquitination and degradation.

## Background

Breast cancer is one of the most common malignancies in women worldwide. According to the latest statistical reports, more than 1.6 million breast cancer new cases are diagnosed each year, which account for 20% of all women cancers [1]. According to the molecular subtype classification, breast cancer can be classified into five groups: Luminal A type, Luminal B type, normal-like, HER2 type and basal-like [2]. Both Luminal A and B types are estrogen receptor alpha positive, which accounts for 70% of all breast cancers. Besides surgical treatment, the adjuvant therapy, such as endocrine therapy, is the most common treatment for luminal type of breast cancer patients [3]. Even the luminal type breast cancer patients could benefit from endocrine therapy, many patients will develop tamoxifen resistance [4]. Thus it is urgent and necessary to develop more novel therapeutics targets for luminal type breast cancer patients.

The P53 protein was firstly found in 1979 and was initially recognized as an oncogene due to the high mutation rate in human cancer [5]. Further studies revealed P53 was an important tumor suppressor gene and critical safeguard for DNA damage, cell stress and malignant transformation process [6, 7]. P53 is composed of 393 amino acids and could be divided into three functional domains, including transcriptional activation domain, DNA binding domain and tetramerization domain [8]. The P53 protein could be activated in several conditions, such as DNA damage and oxidative stress. If it is activated, the P53 half-life will increase, which lead to enhanced activation of P53 target genes, including P21, BAX and Fas [9]. In the meantime, the activation of P53 will lead to G1-S cell cycle arrest, activation of DNA repair process or cell apoptosis. Based on the importance of P53 protein, P53 field is one of the most extensively studied genes in the cancer area. Re-activation of P53 protein is a plausible approach for cancer treatment [10].

The regulation of P53 is tightly controlled via several mechanisms, among which the post-translational modification is one of the most critical manners affecting P53 signaling activity [11–13]. In the unstressed condition, P53 is subject to continuous ubiquitination and proteasome degradation. Several E3 ubiquitin ligases were reported to promote P53 protein ubiquitination and degradation, while the most studied of these are MDM2 protein. The MDM2 protein interacts with P53 at the N-terminal and facilitates P53 ubiquitination and degradation. On the other hand, P53 could locate at the promoter region of MDM2 and facilitate MDM2 gene expression [14]. Besides, the cross regulation between MDM2 and P53, a series of RING family proteins were found to modify P53 poly-ubiquitination and degradation, which were recognized to be involved in the carcinogenic process by suppressing P53 signaling [15–17].

TRIM3 (Tripartite Motif Containing 3) belongs to the RING family proteins, which was firstly reported to associate with myosin and facilitate the target proteins transportation in cells [18]. Further studies reported that TRIM3 could function as a tumor suppressor in several cancers [19–21]. Here, our studies showed that TRIM3 correlated with good prognosis in breast cancer, but related to poor survival only in P53 wild type breast cancer patients. Molecular biology studies showed that TRIM3 promoted P53 degradation and suppressed P53 target gene expression, which ultimately promoted cancer cell growth and inhibited cisplatin-induced apoptosis in P53 wild type breast cancer cells.

## Materials and methods

### Cell culture

MCF-7 and HEK293 cells were ordered form American Type Culture Collection (ATCC). MCF-7 and HEK293 cells were cultured in Dulbecco’s Modified Eagle’s Medium that contains 4.5 g/L glucose and 4 mM L-glutamine (DMEM, 41965, Life Technologies) supplemented with 10% Fetal Bovine Serum (FBS, 10270, Life Technologies). All cell lines were subject to cell line authentication. The cell line authentication via Short Tandem Repeat (STR) was performed via PowerPlex 21 system. The STR data of HEK293 and MCF-7 lines were found consistent with STR data in ATCC.

### Plasmids and siRNA

The Flag-TRIM-3 plasmid was acquired from Origene. The HA-K48 and Ub wild type plasmids were acquired from our previous study [22]. The P53 plasmid was acquired form previous studies [16]. The TRIM3 and P53 deletion variants were sub-cloned from the original plasmids. The Lipofectamin 2000 (1662298, Invitrogen) was used for the plasmids transfection. Small interfering RNAs were used for specific gene knocking-down. The TRIM3 siRNA sequences were: CAAACGAAAGGACAACCCAdTdT; UGGGUUGUCCUUUCGUUUGdTdT and GCAACAACCAGUGUAUUCAdTdT; UGAAUACACUGGUUGUUGCdTdT. The negative control siRNA sequences were: UUCUCCGAACGUGUCACGUTT; ACGUGACACGUUCGGAGAATT. The RNAiMAX reagent (13778150, invitrogen) was used for siRNA transfection.

### RNA extraction and qPCR analysis

RNeasy plus mini kits were used to extract total RNA (Qiagen). Real-time PCR was performed as previously described [23]. 36B4 was used for internal control. The primer sequences were shown here. 36B4: F: GGCGACCTGGAAGTCCAACT; R: CCATCAGCACCACAGCCTTC. P21: F: GTGGCTCTGATTGGCTTTCTG; R: CTGAAAACAGGCAGCCCAAG. BTG2: F: AGACGAGGCAAAGCGGTAAA; R: TCCAACCATTCACGGTCAGA. P53INP1: F: TATGCTGCCCATTTCATTT; R: CTGTGCATAACTCCTGCCCT.

### Quantification of cell viability

MCF-7 cells were transfected with siTRIM3 or siControl into 24-well plates. Twenty-Four hours after transfection, the cells number was countered and 4000 cells were seeded into 96-well plates. The relative cell viability was measured at indicated time points. Cell numbers were determined using the WST-1 cell proliferation reagent as previously described.

### EdU staining assay

For ethynly-deoxyuridine (EdU) labeled DNA, cells were incubated with EdU for 2 h. Later on, the cells were fixed in cell culture plates with 4% formalin. The EdU positive cells were counted with statistical analysis.

### Flow cytometry assay

For the cell cycle analysis, the MCF-7 cells were transfected with 50 uM siTRIM3 or siControl. After 24 h, cells were fixed via 70% ethanol and stained with propidium iodide. For the apoptosis assay, the MDAMB175 cells were transfected with 50 uM siTRIM3 or siControl. Twenty-four hours post-transfection, cell were treated with 10 uM cisplatin for 8 h. Aftern 24 h, cells were stained with propidium iodide and annexin V. The BD LSR flow was used to measure the fluorescence intensity.

### Western blotting

Cells were harvested and lysed with RIPA buffer. Proteins were separated by electrophoresis on SDS–polyacrylamide gel electrophoresis (PAGE) and electro-transferred to PVDF membrane. The antibodies used in this study were listed here: Anti-TRIM3 (HAP043396, Sigma); Anti-P53 (SC-126, Santa Cruz); Anti-Cleaved Caspase-3 (ab2302, Abcam); Anti-HA (MMS-101R, COVANCE); Anti-myc (9E10, ab32, Abcam); Anti-myc (Ab9106, Abcam); Anti-Actin (GB12001, Servicebio). Membranes were then washed with PBS for three times and incubated with secondary antibodies Peroxidase-Conjugated AffiniPure Goat Anti-Mouse IgG or Goat Anti-Rabbit IgG. Fluorescent signals were visualized with ECL system. (amersham imager 600, USA).

### Co-immunoprecipitation assay

Immunoprecipitation was performed as described in previous study [23]. The MCF-7 cells total cell lysis was pre-cleared with rabbit IgG for 2 h and subsequently immunoprecipitated with TRIM3 antibody (HAP043396, Sigma) over night, while rabbit IgG (Santa Cruz) was used as the negative control. The bounded protein was analyzed by Anti-P53 antibody (SC-126, Santa Cruz). For the domain CoIP assay, the P53 or TRIM3 variants were transfected into HEK293 cells. The total cell lysis was pre-cleared with rabbit IgG for 2 h and subsequently immunoprecipitated with Myc antibody overnight, while the rabbit IgG was used as the negative control. The bounded protein was analyzed via GFP antibody (Ab290, Abcam).

### Protein stability assays

About 10^5^ MCF-7 cells were seeded into twenty-four well plates and transfected with 50 uM TRIM3 siRNA or siControl. After 48 h, cells were treated with 100uM cycloheximide (C7698, Sigma) for indicated time points. Samples were subject to western blot for P53 degradation.

### Poly-ubiquitination detection assay

To directly detect the enriched K48-ubiquitinated and total ubiquitinatoin P53 from the cell extracts, HEK293 cells were transfected with 0.8 ug K48 Ubi or 0.8 ug Ub plasmids together with 0.8 ug GFP-P53 plasmid and 0.8 ug Myc-TRIM3 or Myc-vector. After 24 h, the cells were treated with 20 uM MG132 for 7 h,then total protein was extracted and pre-cleared with 30ul protein A (santa cruz, SC-2001) for 4 h. The supernatant was collected and immunoprecipitated by P53 antibody. Western blot with HA antibody was performed to detect total or K48 poly-ubiquitinated P53.

### Immunofluorescence assay

MCF-7 cells were fixed with 4% paraformaldehyde in PBS for 10 min, permeabilized with 0.2% Triton X-100 for 5 min, and blocked by 5% BSA in PBS for 1 h. A rabbit anti-TRIM3 polyclonal antibody (HAP043396, Sigma) and mouse anti-P53 monoclonal antibodies (SC-126, Santa Cruz) were used, followed by Alexa Flour 647 (Invitrogen) anti-rabbit antibody and FITC-conjugated anti-mouse antibodies (Jackson ImmunoResearch, West Grove, PA). As negative controls, the samples were incubated with the secondary antibodies without primary antibodies. Images were acquired under conditions fulfilling the Nyquist criterion using Nikon A + laser scanning confocal system with a 60X oil NA1.4 objective and pinhole size of 1.0 Airy Unit. The acquired pictures were further processed and assembled using ImageJ.

### Statistics

Student's *t*-test, Pearson correlation coefficient, and Cox regression analysis were used for comparisons. A *P*-value of < 0.05 was considered to be significant.

## Results

### TRIM3 negative correlates P53 signaling in breast cancer cells

**Fig. 1:**
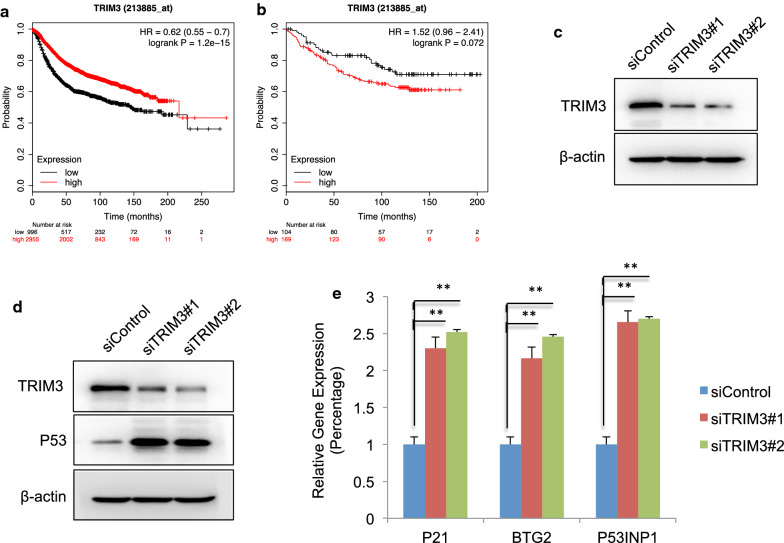
TRIM3 negatively correlates P53 signaling in breast cancer cells. **a** The KMPLOT online analysis showed that TRIM3 related to good prognosis in all breast cancer patients. **b** The KMPLOT online analysis showed that TRIM3 tend to relate to good prognosis in P53 wild type breast cancer patients. **c** TRIM3 knocking down efficiency in MCF-7 cells. MCF-7 cells were transfected with TRIM3 siRNA. After 48 h, TRIM3 protein was determined by Western blot. Actin was used as internal control. **d** TRIM3 depletion increased P53 protein levels in MCF-7 cells. MCF-7 cells were transfected with siControl or siTRIM3. After 48 h, cells were harvested for western blot analysis. TRIM3 and P53 protein levels were determined by Western blot. Actin was used as internal control. **e** TRIM3 depletion increased P53 target gene expression in MCF-7 cells. MCF-7 cells were transfected with siControl or siTRIM3. After 48 h, total RNA was extracted for gene expression analysis. Each group was done in triplicates. *P < 0.05; **P < 0.01; ***P < 0.001 for target gene expression comparison

We firstly analyzed the prognostic effect of TRIM3 in breast cancer sample. From the public available database (https://kmplot.com/analysis), we observed that TRIM3 was correlated with good overall survival in all breast cancer patients, which was consistent with previous reported role of TRIM3 in other cancers (Fig. 1a) [19–21]. However, TRIM3 related to poor prognosis especially in P53 WT breast cancer groups (Fig. 1b). Further analysis showed that TRIM3 was elevated in TP53 wild type group in breast cancer cells, but failed to correlate with P53 target gene expression, nor prognosis in both P53 WT and mutant groups (Additional file 1: Fig. S1a–e). We further utilized MCF-7 cell, a breast cancer cell line with WT P53, as a model to carry out the cell biology studies. TRIM3 was depleted via two independent siRNAs (Fig. 1c). Immuno-blotting showed that TRIM3 depletion increased the P53 protein level in MCF-7 cells (Fig. 1d). Besides, real-time PCR assay showed that TRIM3 depletion increased classical P53 signaling target gene expression, such as CDKN1A, BTG2 and P53INP1 (Fig. 1e).

### TRIM3 depletion activates P53 signaling, inhibits cell growth and promotes apoptosis in breast cancer cells

**Fig. 2:**
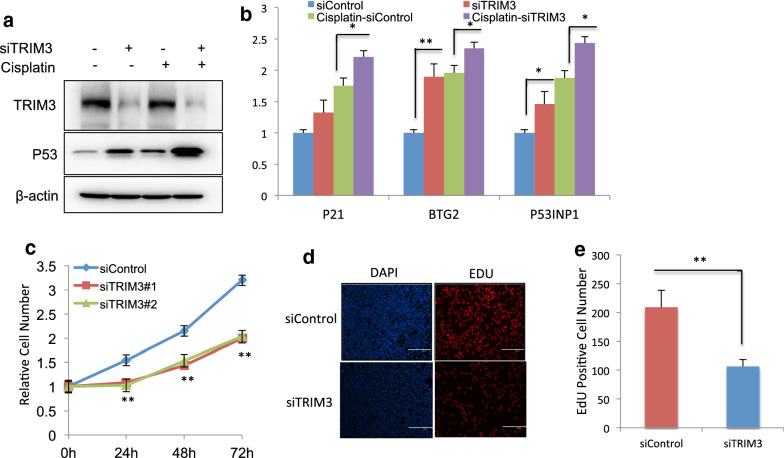
TRIM3 depletion activates P53 signaling, inhibits cell growth in MCF-7 breast cancer cells. **a** TRIM3 depletion increased P53 protein levels in both vehicle and cisplatin-treated conditions. MCF-7 cells were transfected with siControl or siTRIM3. 1 ug/ml Cisplaitn was added to treat the cells for 6 h. Then cells were harvested for western blot analysis. TRIM3 and P53 protein levels were determined by Western blot. Actin was used as internal control. **b** TRIM3 depletion increased P53 target gene expression in both vehicle and cisplatin-treated conditions. MCF-7 cells were transfected with siControl or siTRIM3. After 48 h, 1 ug/ml Cisplaitn was added to treat the cells for 6 h. total RNA was extracted for gene expression analysis. Each group was done in triplicates. *P < 0.05; **P < 0.01; ***P < 0.001 for target gene expression comparison. **c** TRIM3 depletion inhibited MCF-7 cells proliferation. MCF-7 cells were transfected with siControl or siTRIM3. After 24 h, the WST assay was used to determine the cellar metabolic activity at indicated time points after infection. Experiments were done in triplicates. *P < 0.05; **P < 0.01; ***P < 0.001 for cell growth comparison. **d**, **e** TRIM3 depletion inhibited the number of EdU positive breast cancer cells. MCF-7 cells were transfected with siControl or siTRIM3. After 24 h, EdU was added into the medium for 2 h incubation. The absolute cell number was counted to indicate cell proliferation activity

**Fig. 3:**
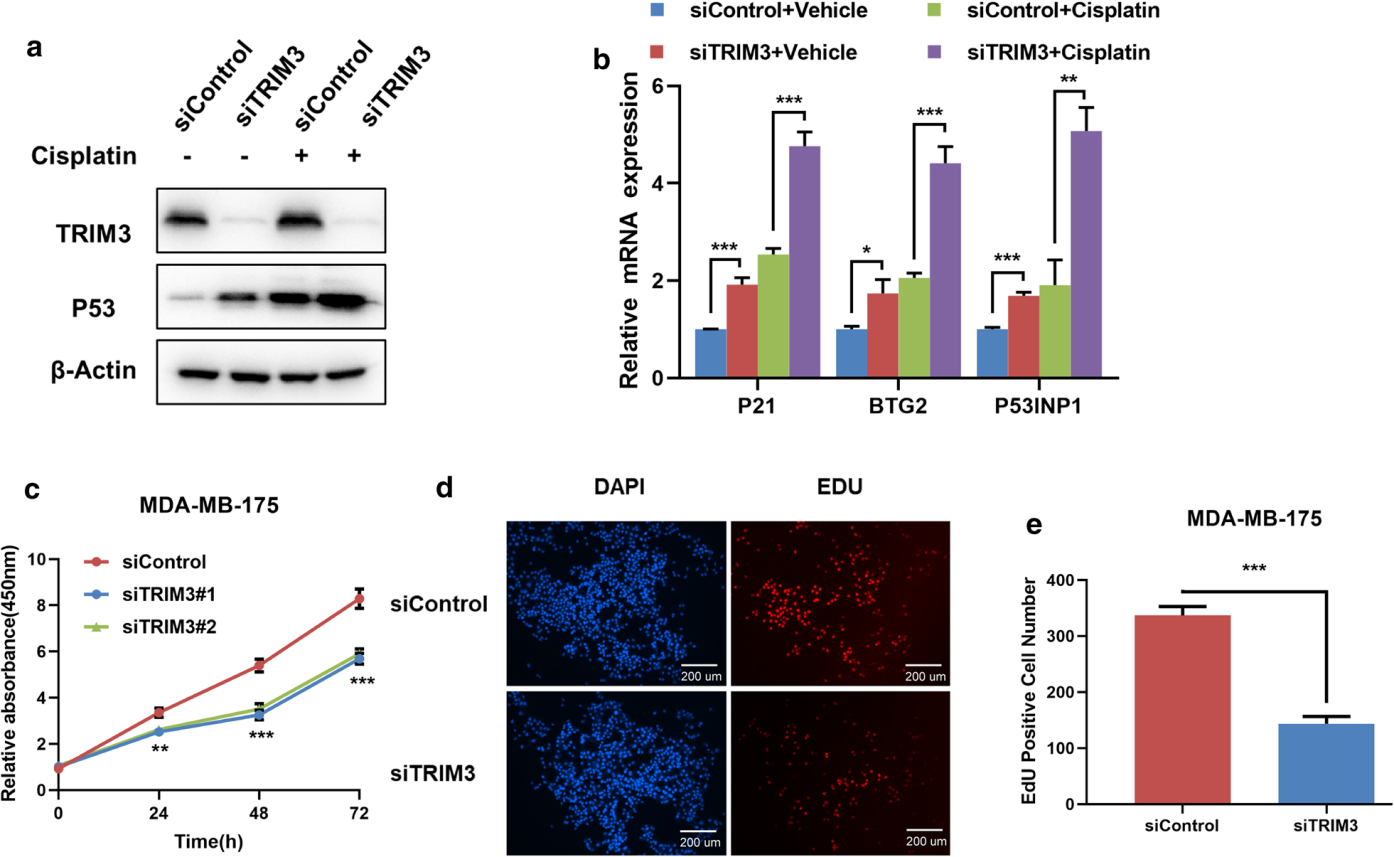
TRIM3 depletion activates P53 signaling, inhibits cell growth in MDAMB175 breast cancer cells. **a** TRIM3 depletion increased P53 protein levels in both vehicle and cisplatin-treated conditions. MDAMB175 cells were transfected with siControl or siTRIM3. 1 ug/ml Cisplaitn was added to treat the cells for 6 h. Then cells were harvested for western blot analysis. TRIM3 and P53 protein levels were determined by Western blot. Actin was used as internal control. **b** TRIM3 depletion increased P53 target gene expression in both vehicle and cisplatin-treated conditions. MDAMB175 cells were transfected with siControl or siTRIM3. After 48 h, 1 ug/ml Cisplaitn was added to treat the cells for 6 h. total RNA was extracted for gene expression analysis. Each group was done in triplicates. *P < 0.05; **P < 0.01; ***P < 0.001 for target gene expression comparison. **c** TRIM3 depletion inhibited MDAMB175 cells proliferation. MDAMB175 cells were transfected with siControl or siTRIM3. After 24 h, the WST assay was used to determine the cellar metabolic activity at indicated time points after infection. Experiments were done in triplicates. *P < 0.05; **P < 0.01; ***P < 0.001 for cell growth comparison. **d**, **e** TRIM3 depletion inhibited the number of EdU positive breast cancer cells. MDAMB175 cells were transfected with siControl or siTRIM3. After 24 h, EdU was added into the medium for 2 h incubation. The absolute cell number was counted to indicate cell proliferation activity

**Fig. 4:**
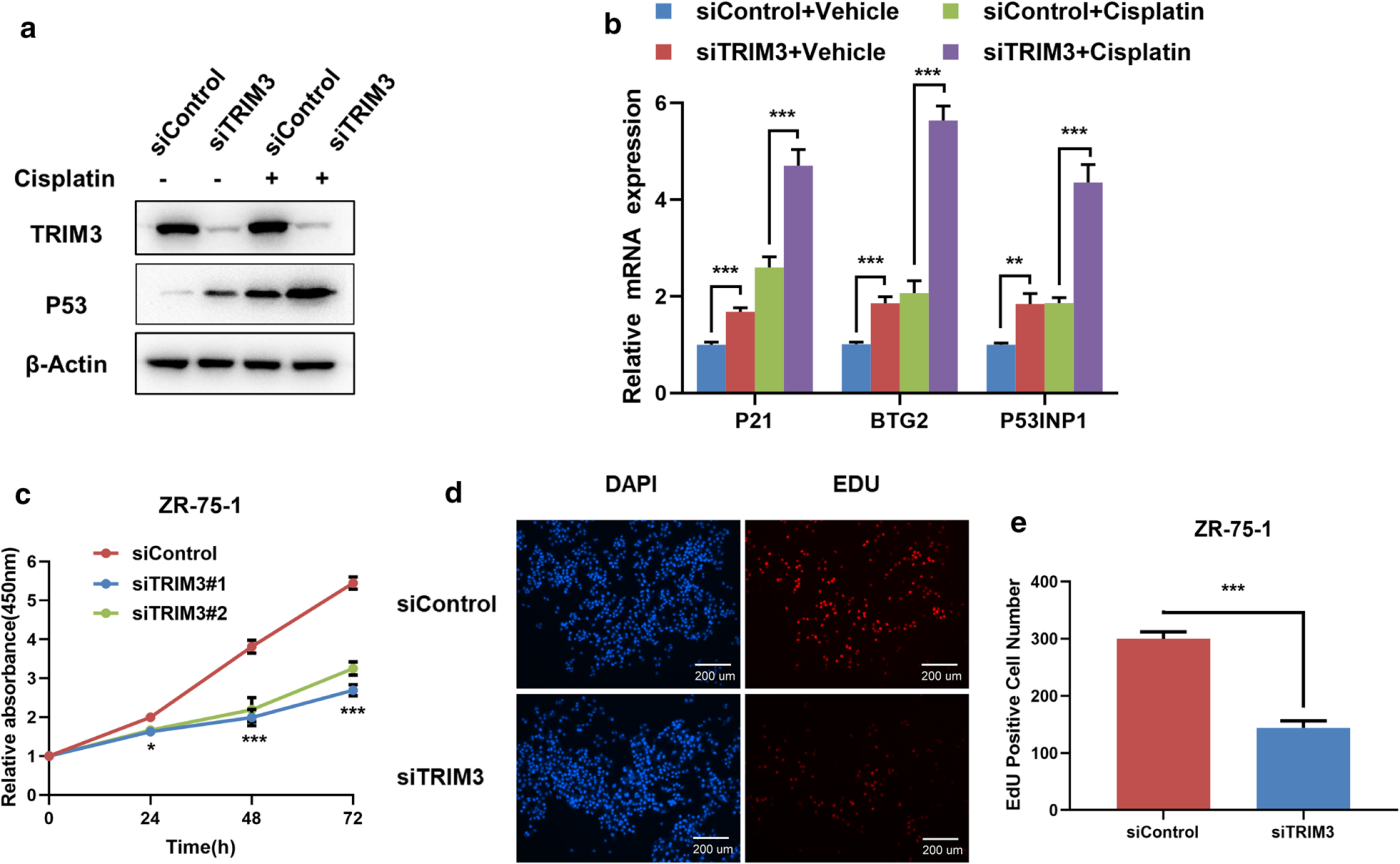
TRIM3 depletion activates P53 signaling, inhibits cell growth in ZR751 breast cancer cells.** a** TRIM3 depletion increased P53 protein levels in both vehicle and cisplatin-treated conditions. ZR751 cells were transfected with siControl or siTRIM3. 1 ug/ml Cisplaitn was added to treat the cells for 6 h. Then cells were harvested for western blot analysis. TRIM3 and P53 protein levels were determined by Western blot. Actin was used as internal control. **b** TRIM3 depletion increased P53 target gene expression in both vehicle and cisplatin-treated conditions. ZR751 cells were transfected with siControl or siTRIM3. After 48 h, 1 ug/ml Cisplaitn was added to treat the cells for 6 h. total RNA was extracted for gene expression analysis. Each group was done in triplicates. *P < 0.05; **P < 0.01; ***P < 0.001 for target gene expression comparison. **c** TRIM3 depletion inhibited ZR751 cells proliferation. ZR751 cells were transfected with siControl or siTRIM3. After 24 h, the WST assay was used to determine the cellar metabolic activity at indicated time points after infection. Experiments were done in triplicates. *P < 0.05; **P < 0.01; ***P < 0.001 for cell growth comparison. **d**, **e** TRIM3 depletion inhibited the number of EdU positive breast cancer cells. ZR751 cells were transfected with siControl or siTRIM3. After 24 h, EdU was added into the medium for 2 h incubation. The absolute cell number was counted to indicate cell proliferation activity

In order to test the effect of TRIM3 in both normal and P53-acivated conditions, we utilized cisplatin, one chemotherapy drug, to activate P53 pathway in breast cancer cells. The immuno-blotting showed that TRIM3 could increase P53 protein level in both vehicle and cisplatin-treated conditions (Fig. 2a). Besides, Q-PCR results indicated that TRIM3 depletion increased the P53 target gene expression in both vehicle and cisplatin-treated conditions (Fig. 2b). The WST-1 assay showed that TRIM3 knocking down inhibited breast cancer cell proliferation (Fig. 2c), while the EdU staining assay also indicated TRIM3 depletion significantly decreased the cell numbers of EdU incorporation (Fig. 2d, e). We further tested the TRIM3 depletion effect in another two breast cancer cell lines with P53 WT. The data showed that TRIM3 depletion increased P53 protein level and P53 target gene expression in both vehicle and cisplatin-treated conditions (Figs. 3a, b and 4a, b). Besides, TRIM3 depletion inhibited cell proliferation in both of the two cell lines (Figs. 3c–e and 4c–e).

**Fig. 5:**
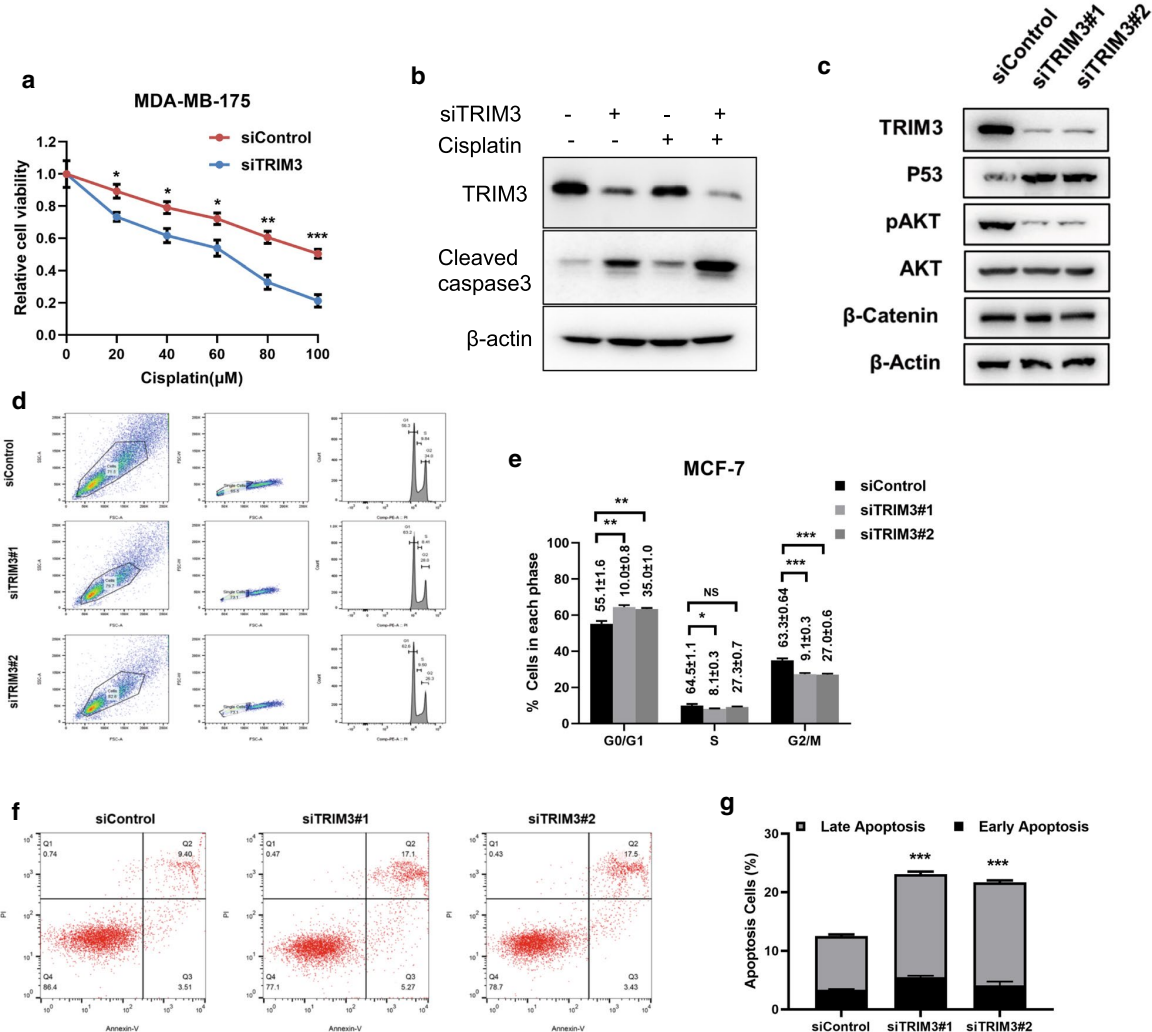
TRIM3 depletion promoted cell apoptosis and cell cycle arrest in breast cancer cells.** a** TRIM3 depletion sensitized cisplatin-mediated cell inhibition in MDAMB175 cells. MDAMB175 cells were transfected with 50 uM siTRIM3 or siControl. After 24 h, cells were treated with cisplatin for indicated concentration for 24 h. The cell viability was determined via CCK8 assay. **b** TRIM3 depletion increased cleaved caspase 3 protein levels in both vehicle and cisplatin-treated conditions. MDAMB175 cells were transfected with siControl or siTRIM3. 1 ug/ml Cisplaitn was added to treat the cells for 6 h. Then cells were harvested for western blot analysis. TRIM3 and cleaved caspase 3 protein levels were determined by Western blot. Actin was used as internal control. **c** TRIM3 depletion increased phosphorylation level of AKT in MDAMB175 cells. MDAMB175 cells were transfected with siControl or siTRIM3. After 24 h, cells were harvested for western blot analysis. TRIM3, P53, AKT, phspho-AKT, b-catenin protein levels were determined by Western blot. Actin was used as internal control. **d**, **e** TRIM3 depletion caused G1 cell cycle arrest in MCF-7 cells. MCF-7 cells were transfected with siControl or siTRIM3. After 24 h, cells were fixed and stained with PI. The relative proportion of cell cycle in each group was determined via FACS analysis. Each group was done in triplicates. *P < 0.05; **P < 0.01; ***P < 0.001 for comparison. **f**, **g** TRIM3 depletion promoted apoptosis in MDAMB175 cells. MDAMB175 cells were transfected with siControl or siTRIM3. After 24 h, cells were stained with PI and Annexin V. Then cells were subject to FACS analysis for the proportion of apoptotic cells. Each group was done in triplicates. *P < 0.05; **P < 0.01; ***P < 0.001 for comparison

Since P53 is an important regulator in cell apoptosis and cell cycle, we carried out more experiments to measure TRIM3 role in cell apoptosis and cell cycle. The cell apoptosis assays were carried out mostly in MDAMB175 cells, since the apoptotic signaling is defect in MCF-7 cells. In Fig. 5a, TRIM3 depletion could significantly sensitize cisplatin-induced cell death in MDAMB175 cells. The immuno-blotting showed that TRIM3 depletion could increase cleaved-caspase 3 level, but also phospho-AKT level (Fig. 5b, c). The cell cycle analysis indicated that TRIM3 depletion could induce G1 cell cycle arrest in MCF-7 cells (Fig. 5d, e). The PI/Annexin V double staining showed that TRIM3 depletion promoted cell apoptosis in MDAMB175 cells (Fig. 5f, g).

### TRIM3 is localized in the cytosol and interact with P53 in breast cancer cells

**Fig. 6:**
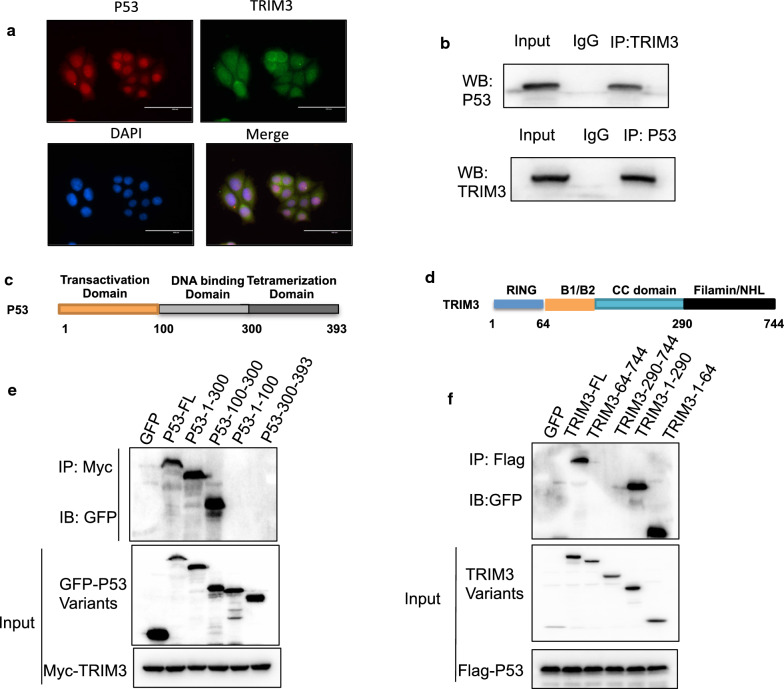
TRIM3 is localized in the cytosol and interact with P53 in breast cancer cells.** a** Intracellular localization analysis of P53 and TRIM3 by immunofluorescence assay. MCF-7 cells were cultured in normal medium before fixation. Intracellular localization of TRIM3 (green) and P53 (red) were shown. Nuclei (blue) were stained with 4′,6-diamidino-2-phenylindole (DAPI). **b** Co-IP assay revealed association between endogenous P53 and TRIM3 protein in MCF-7 cells. MCF-7 cells were harvested with RIPA lysis buffer. CO-IP was performed using antibody as indicated. **c** P53 domain structure and deletion variants in the study. **d** TRIM3 domain structure and deletion variants in the study. **e** P53 DNA binding domain is required to mediate the TRIM3-P53 interaction. HEK293 cells were transfected with myc-TRIM3 together with GFP-P53 full length or variants. After 24 h, cells were harvested with NP-40 lysis buffer. CoIP was performed and the possible interacted P53 variants were detected by GFP antibody. **f** RING domain of TRIM3 is required to mediate the TRIM3-P53 interaction. HEK293 cells were transfected with Flag-P53 together with GFP-TRIM3 full length or variants. After 24 h, cells were harvested with NP-40 lysis buffer. CoIP was performed and the possible interacted TRIM3 variants were detected by GFP antibody

We further analyzed the localization of TRIM3 and P53 in breast cancer cells. Immuno-staining showed that TRIM3 was mainly localized in the cytoplasm, while P53 was localized mostly in the nuclear (Fig. 6a). The endogenous immono-precipitation indicated that TRIM3 could associate with P53 in breast cancer cells (Fig. 6b). We further investigated the interaction domain of P53 with TRIM3. P53 is composed of three functional domains: transactivation domain, DNA binding domain and tetramerization domain (Fig. 6c), while TRIM3 is composed of four functional domains: RING domain, B1/B2 domain, CC domain and Filamin/NHL domain (Fig. 6d). We sub-cloned the variants of TRIM3 and P53. Further immuno-precipitation showed that the DNA binding domain was required for P53 to associate with TRIM3, while TRIM3 interacted with P53 via its RING domain (Fig. 6e, f).

### TRIM3 facilitated P53 K48-linked poly-ubiquitination and degradation

**Fig. 7:**
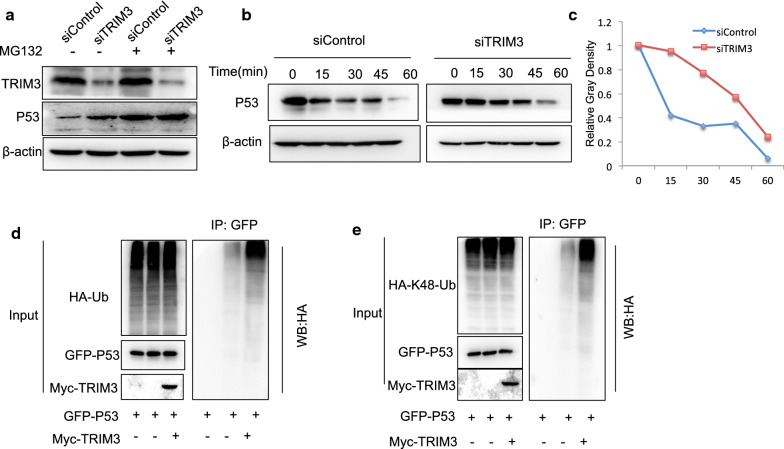
TRIM3 facilitated P53 K48-linked poly-ubiquitination and degradation.** a** In the presence of the proteasome inhibitor MG132, the degradation effect of TRIM3 on P53 did not further increase P53 protein levels. MCF-7 cells were transfected with with siControl or siTRIM3. After 24 h, cells were treated with 20 uM MG132/vehicle for 7 h. Cell lysates were prepared for Western blot analysis. The results are representative for three independent experiments. **b**, **c** TRIM3 decreased P53 half-life in MCF-7 cells. MCF-7 cells were transfected with with siControl or siTRIM3. After 24 h, cells were treated with 100 µM cycloheximide/vehicle for indicated times. Cell lysates were prepared for Western blot analysis. The results are representative for three independent experiments. The P53 relative density was measured by Image J software. **d** TRIM3 increased the overall poly-ubiquitination of P53. HEK293 cells were transfected with 1 µg GFP-P53 together with 0.5 µg Myc-TRIM3 or Myc vector. After 24 h, cells were transfected with 1 µg HA-Ub plasmid. After another 24 h, the cell extracts were immunoprecipitated with HA antibody. The poly-ubiquitinated P53 was detected via western blotting analysis. **e** TRIM3 increases K48-linked poly-ubiquitination of P53. HEK293 cells were transfected with 1 µg GFP-P53 together with 0.5 µg Myc-TRIM3 or Myc vector. After 24 h, cells were transfected with 1 µg HA-K48-Ubi plasmid. After another 24 h, the cell extracts were immunoprecipitated with HA antibody. The K48-linked poly-ubiquitinated P53 was detected via western blotting analysis

Since TRIM3 was shown to interact with P53 in breast cancer cell, we further investigated the potential molecular mechanisms. TRIM3 depletion could increase P53 protein level, which effect could be diminished via the proteasome inhibitor MG132 (Fig. 7a). This might indicated that TRIM3 modulated P53 via protein stability. Then we utilized cycloheximide, the protein synthesis inhibitor, to measure the protein stability. Figure 4b showed that TRIM3 depletion significantly increased P53 half-life (Fig. 7b, c). The ubiquitin-based immuno-precipitation assay showed that TRIM3 could facilitate the overall ubiquitination level of P53 (Fig. 7d). In order to investigate the ubiquitin manner of TRIM3 on P53 protein, the K48 specific Ubiquitin plasmid were transfected to test the ubiquitination manner. The ubiquitin-based immuno-precipitation assay showed that the presence of TRIM3 could significantly promote K48-linked ubiquitination of P53 (Fig. 7e).

## Discussion

**Fig. 8:**
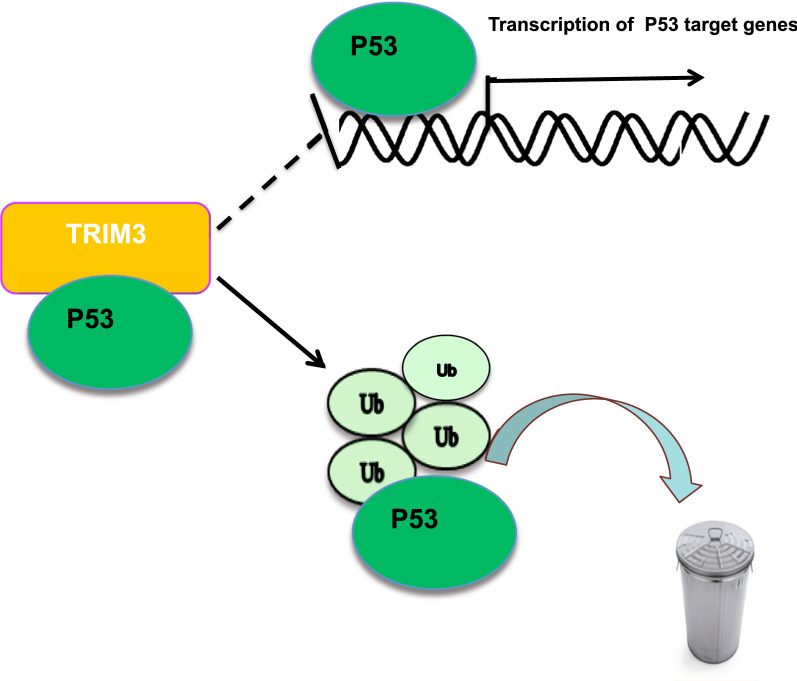
The hypothetical model for TRIM3 regulating P53 pathway in P53 wild type breast cancer: TRIM3 protein associated with P53 and promoted P53 degradation via inducing P53 K48-linked poly-ubiquitination

In the study, we demonstrated that the RING family protein TRIM3 interacted with P53 protein via its RING domain, promoted P53 K48-linked poly-ubiquitination and degradation in breast cancer cells (Fig. 8). Furthermore TRIM3 depletion induced activation of P53 signaling, cell growth arrest and cisplatin-induced apoptosis, which could be a promising therapeutic target for P53 WT breast cancer patients.

The importance of P53 in cancer has been investigated for 30 years, based on the fact that P53 is one of the most frequently mutated tumor suppressor genes in human cancer [5]. The mutated P53 could accumulate in the nuclear and disturb the assembly of DNA repair complex and facilitate tumor progression. Besides, mutated P53 could also act as an oncogene with new activities, termed “gain-of-function”, that contribute to apoptosis resistance, cancer invasion and so on [24]. However, the mutation rate of P53 is approximately 30% of all breast cancer patients [25]. Interestingly, there is a reverse correlation between ER alpha positivity and P53 mutation. The P53 mutation rate is 20% in ER alpha positive samples, while high up to 89% of P53 mutation in ER alpha negative breast cancer samples [26]. This might indicated that P53 could exert its tumor suppressor role in ER alpha positive breast cancer, which also give a possible explanation why ER alpha positive breast cancer patients have better prognosis compared with ER alpha negative ones [2]. Besides, the P53 mutations in breast cancer, P53 is also subject to several other kinds of function suppressions. For example, ER alpha could interact with P53 protein and suppress P53 target gene expression [27]. Besides, P53 expression level was decreased in breast cancer either by promoter hyper-methylation or by post-translational modification [28].

P53 is one stress-related protein with the half-life approximately 20 min [29]. The P53 signaling activity is precisely control mainly through ubiquitination and proteasome-dependent degradation [30]. Several E3 ubiquitin ligases have been shown to directly promote P53 poly-ubiquitination and degradation, including MDM2 and CHIP [14, 15]. MDM2 is the most studied E3 ubiquitin ligase for P53 signaling. MDM2 facilitates poly-ubiquitination at several lysine residues in P53 protein and induces P53 degradation, while P53 could locate at the promoter region of MDM2 and facilitate MDM2 gene expression. However, our current study reported a novel E3 ligase TRIM3, a cytoplasmic protein, which promotes P53 ubiquitination in K48-linked manner and degradation. Interestingly TRIM3 could interact with P53 protein, which means suppression of TRIM3 expression or small chemical compounds interfering the TRIM3-P53 interaction could be a promising strategy to restore P53 signaling activity in breast cancer cells.

TRIM3 belongs to the group of tripartite motif family and is composed of zinc-binding domain, RING domain, B1/2 domain and coiled region [31]. TIRM3 was firstly reported as the partner of myosin and facilitate the transportation of the target proteins [18]. TRIM3 were reported to be decreased in several human cancers, such as gastric cancer, colon cancer and liver cancer [19, 31–33]. However, although TRIM3 was shown to function as a tumor suppressor gene in several cancer types, we did not observe any dramatic TRIM3 level change in breast cancer compared with normal breast tissue, which might indicated the uncertain role or dual roles of TRIM3 might exist in breast cancer. Our molecular study revealed that TRIM3 could suppress P53 signaling, facilitate cell growth and resistance to cisplatin-induced cell apoptosis. Our finding indicates TRIM3 plays a oncogenic role in P53 WT breast cancer cells, which is opposite to previous studies [19, 31]. This interesting finding not only increases the understanding of P53 post-translational modifications but also implicates the multi-face of TRIM3 in different cancer background.

In conclusion, our study demonstrates the E3 ligase TRIM3 as a regulator of P53 signaling in human breast cancer cells. TRIM3 suppresses P53 protein level and promotes breast cancer cell growth and anti-apoptosis. As a newly discovered modulator of P53 pathway, TRIM3 could be a promising target to treat P53 WT breast cancer.

## Conclusion

Our study demonstrates the E3 ligase TRIM3 as a regulator of P53 signaling in human breast cancer cells. TRIM3 suppresses P53 protein level and promotes breast cancer cell growth and anti-apoptosis. As a newly discovered modulator of P53 pathway, TRIM3 could be a promising target to treat P53 WT breast cancer.

## Supplementary information

**Additional file 1**. Supplementary Figures.

## Abbreviations

ER alpha: Estrogen receptor alpha
RING: Really interesting new gene
TRIM: Tripartite Motif Containing
EdU: Ethynly-deoxyuridine
MDM2: Mouse double minute 2
