# Supplementary figures and images for: TRIM3 inhibits P53 signaling in breast cancer cells
Source: Cancer Cell Int. 2020 Nov 23;20:559. doi: 10.1186/s12935-020-01630-z (PMC7685606; doi:10.1186/s12935-020-01630-z)

Supplementary Figure 1

A

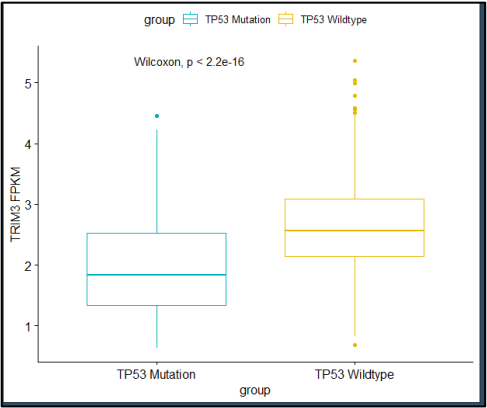

B

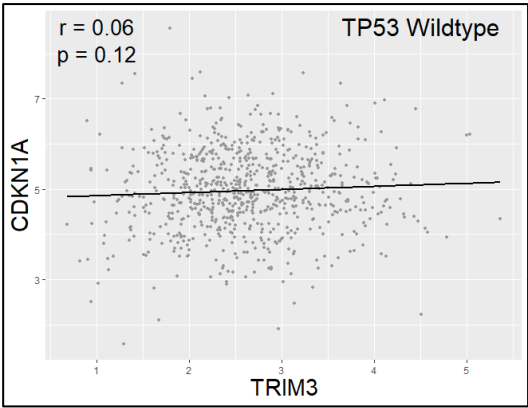

C

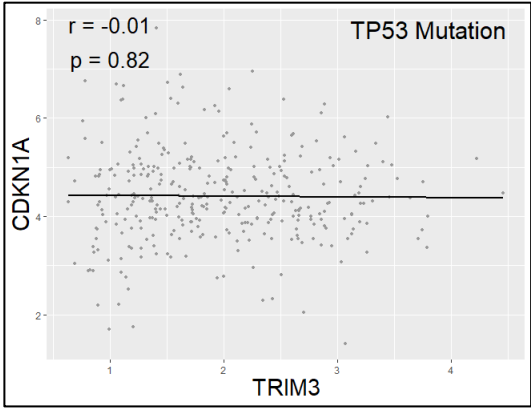

D

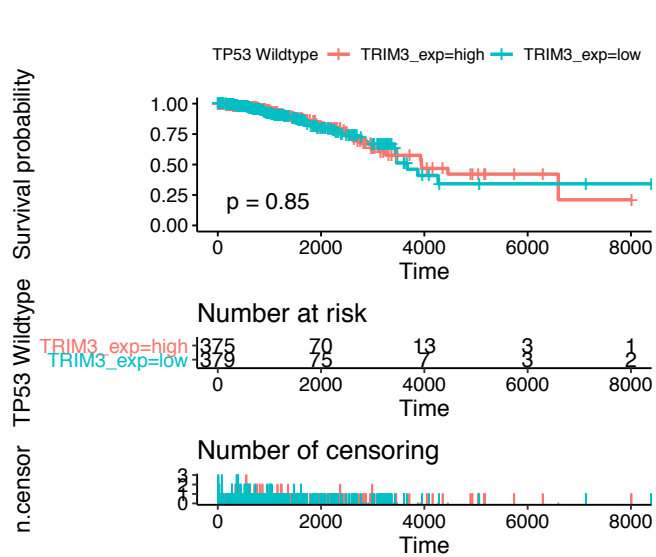

E

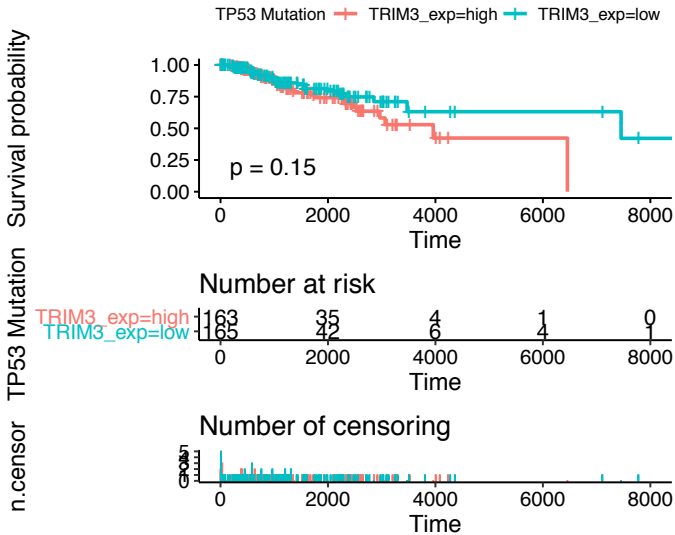

Supplement: Supplementary file 1 — Additional file 1. Supplementary Figures. [file 12935_2020_1630_MOESM1_ESM.pdf]
